# A Hybrid Model for Family History Information Identification and Relation Extraction: Development and Evaluation of an End-to-End Information Extraction System

**DOI:** 10.2196/22797

**Published:** 2021-04-22

**Authors:** Youngjun Kim, Paul M Heider, Isabel RH Lally, Stéphane M Meystre

**Affiliations:** 1 Biomedical Informatics Center Medical University of South Carolina Charleston, SC United States; 2 Department of Computer Science College of Charleston Charleston, SC United States

**Keywords:** natural language processing, machine learning, deep learning, named entity recognition, clinical entity identification, relation extraction

## Abstract

**Background:**

Family history information is important to assess the risk of inherited medical conditions. Natural language processing has the potential to extract this information from unstructured free-text notes to improve patient care and decision making. We describe the end-to-end information extraction system the Medical University of South Carolina team developed when participating in the 2019 National Natural Language Processing Clinical Challenge (n2c2)/Open Health Natural Language Processing (OHNLP) shared task.

**Objective:**

This task involves identifying mentions of family members and observations in electronic health record text notes and recognizing the 2 types of relations (family member-living status relations and family member-observation relations). Our system aims to achieve a high level of performance by integrating heuristics and advanced information extraction methods. Our efforts also include improving the performance of 2 subtasks by exploiting additional labeled data and clinical text-based embedding models.

**Methods:**

We present a hybrid method that combines machine learning and rule-based approaches. We implemented an end-to-end system with multiple information extraction and attribute classification components. For entity identification, we trained bidirectional long short-term memory deep learning models. These models incorporated static word embeddings and context-dependent embeddings. We created a voting ensemble that combined the predictions of all individual models. For relation extraction, we trained 2 relation extraction models. The first model determined the living status of each family member. The second model identified observations associated with each family member. We implemented online gradient descent models to extract related entity pairs. As part of postchallenge efforts, we used the BioCreative/OHNLP 2018 corpus and trained new models with the union of these 2 datasets. We also pretrained language models using clinical notes from the Medical Information Mart for Intensive Care (MIMIC-III) clinical database.

**Results:**

The voting ensemble achieved better performance than individual classifiers. In the entity identification task, our top-performing system reached a precision of 78.90% and a recall of 83.84%. Our natural language processing system for entity identification took 3rd place out of 17 teams in the challenge. We ranked 4th out of 9 teams in the relation extraction task. Our system substantially benefited from the combination of the 2 datasets. Compared to our official submission with F_1_ scores of 81.30% and 64.94% for entity identification and relation extraction, respectively, the revised system yielded significantly better performance (*P*<.05) with F_1_ scores of 86.02% and 72.48%, respectively.

**Conclusions:**

We demonstrated that a hybrid model could be used to successfully extract family history information recorded in unstructured free-text notes. In this study, our approach to entity identification as a sequence labeling problem produced satisfactory results. Our postchallenge efforts significantly improved performance by leveraging additional labeled data and using word vector representations learned from large collections of clinical notes.

## Introduction

Family history (FH) information included in the electronic health record (EHR) is important to assess the risk of inherited medical conditions. For certain diseases such as breast cancer [1,2] and colorectal cancer [3,4], FH is an important risk factor. FH information has been recorded in both structured and narrative free text, but often documented only in the latter. Polubriaginof et al [5] reported that free-text notes contained more comprehensive information than structured data. Natural language processing (NLP) has the potential to extract this information from unstructured free-text notes to improve patient care and decision making.

This manuscript describes the end-to-end information extraction (IE) system the Medical University of South Carolina (MUSC) team developed when participating in the 2019 National Natural Language Processing Clinical Challenge (n2c2)/Open Health Natural Language Processing (OHNLP) track on FH extraction [6]. This shared task is built on the BioCreative/OHNLP 2018 FH extraction task [7]. It involves (1) identifying mentions of family members and observations in EHR text notes and (2) recognizing the relations between family members, observations, and living status.

Entity identification and relation extraction are often considered subtasks of IE. The semantic types of concepts of interest have been defined for different target tasks. Named entity recognition (NER) was introduced in the sixth of a series of Message Understanding Conferences [8] and Automatic Content Extraction programs [9]. The goal of NER is to extract and classify proper named or specialized entities into predefined categories [8]. Relation extraction deals with a pair of concepts [10] (ie, binary relations) or higher-order relations, which are *n*-ary relations among *n* typed entities [11]. It aims to determine whether entities are in a relation and how they are semantically related. Medical concept extraction is closely related to our target task and has advanced from the general text NER by sharing the algorithms and features. It has aimed to extract medical information such as disease diagnoses, medications, laboratory data, and appliances from EHR text notes [12-16].

Several studies focusing on FH information have been reported. Goryachev et al [17] created a rule-based system for identifying family members and their related diagnoses. They observed that FH was often mentioned intermixed with the patient's own medical history, making this task challenging. Bill et al [18] developed an NLP system for extracting FH information from History and Physical notes. Their NLP pipeline identified family member and observation entities, relations between them, and attributes such as vital status and age. FH information extraction was the focus of the BioCreative/OHNLP 2018 task [7]. The best performance on this shared task was achieved by Shi et al [19] with F_1_ scores of 89.01% on subtask 1 and 63.59% on subtask 2. They proposed joint modeling of entities and relations by 2 stacked neural networks with shared parameters.

The goal of this study was to extract the health information of patients and their relatives from unstructured EHR notes. Our system aims to achieve a high level of performance in this task by integrating heuristics and advanced information extraction methods. We approach entity identification as a sequence labeling problem. We applied a bidirectional long short-term memory (Bi-LSTM) [20] algorithm, a widely used structured prediction algorithm. The input of the LSTM network included vector representations generated by Embeddings from Language Models (ELMo) [21] contextual embeddings. We hypothesized that applying the LSTM to this problem can yield accurate FH information extraction. Our voting ensemble is created based on the fact that the LSTM algorithm is not deterministic; that is, every time the model is trained, the results vary. The proposed ensemble can provide efficient and convenient integration of individual LSTM models. For relation extraction, we implemented online gradient descent (OGD) [22] models with lexical features.

This study's contribution also includes improved performance on both subtasks by exploiting additional labeled data and clinical text–based embedding models. We added other labeled data used in the previous shared task to the training set. We retrained the classifier using a larger set of training data. We also used word embeddings pretrained with large quantities of clinical text. Our experimental results show that these efforts significantly improve the performance of both subtasks, especially relation extraction.

The following sections describe the details of the 2 subtasks and discuss IE models developed to recognize the entities and their relations from EHRs. We then present the experimental results and investigate the performance improvements resulting from our postchallenge efforts.

## Methods

Our research focuses on the extraction of mentions of family members and related information recorded in EHR text notes. The first subtask, entity identification, involves detecting 2 types of entities: family members and observations. Only relatives in the first degree (eg, ‘*Mother*’ and ‘*Son*’) and second degree (eg, ‘*Grandparent*’ and ‘*Cousin*’) are annotated [7]. Other relatives such as ‘*Spouse*’ and ‘*Nephew*’ are excluded. The normalized name and the side of family are annotated as attributes of each family member. Observation (disease) entities in the family history are also annotated. The second subtask, relation extraction, is to determine the existence of relations between family members and other information (ie, living status or observation). Two types of relations were therefore annotated: family member-living status and family member-observation. For relations between a family member and living status, the score representing the health status of the family member is annotated. Negation information is annotated to indicate whether the observation is negated in the relation between a family member and the associated observation.

### Data Description

Clinical text notes representing patient FH information were selected from the Mayo Employee and Community Health cohort [7]. Table 1 shows the number of annotated entities and relations in the training set. The training set includes 99 clinical notes with 801 family member and 978 observation entities. Living status entities are less common and account for about half of the number of family members. For the observation category, the number of relations is less than the number of entities. This means that some observations are not related to any family member.

**Table 1:** Number of annotated entities and relations in the training set.

Variable	Entities	Relations
Family member	801	N/A^a^
Living status	415	425
Observation	978	753

^a^N/A: not applicable because relations between family members were not annotated.

### Entity Identification Methods

We addressed entity identification with rule-based and machine learning–based approaches. We describe each approach and present a voting ensemble–based method.

#### Rule-Based System for Family Member Entities

Our rule-based system for family member entity recognition uses a sliding window with simple term matching and part-of-speech filtering. We used NLTK [23] (a Python Natural Language Toolkit) to split each note into sentences and then each sentence into tokens annotated with part-of-speech tags. Each token matching a relevant family member term (eg, “*daughter*”, “*son*”, or “*child*”) that was also tagged as a noun (ie, NN, NNP, or NNS) was flagged as a valid mention.

#### Machine Learning–Based Models

We trained sequence labeling models using Bi-LSTM [20,24] to assign a semantic category label to each word in a sequence. Bi-LSTM can combine both forward and backward information of each word.

For this sequence labelling problem, we tokenized the input text. The training data were annotated with BIO token tags (B: beginning, I: inside, or O: outside of an entity; eg, “B-observation” for a token at the beginning of an observation mention). We also included the outputs of the 2 external resources (the 2010 Informatics for Integrating Biology and the Bedside [i2b2] [25] and MetaMapLite [26]) described in the following paragraphs as inputs to the LSTM network. Similar to the word token, the prediction from each external resource was also encoded with BIO tags.

First, we used the medical concept extraction model trained with the 2010 i2b2 challenge data [25]. The training set containing 349 text documents was used to create a Bi-LSTM model that identified medical *problem*, *treatment*, and *test* concepts from the FH extraction task corpus. We also used MetaMapLite [26] (2019 AA version) to identify Unified Medical Language System (UMLS) Metathesaurus concept mentions along with their semantic type. We aligned MetaMap outputs with the entity types of subtask 1 to choose the relevant semantic types. Table 2 lists the 10 most frequently aligned UMLS semantic types used by MetaMap for observation entity extraction. The first and second columns display semantic type names and abbreviations. The third column shows the number of observation entities from the training corpus aligned with each semantic type. The last column shows the mapping probability for each semantic type and observation category. For instance, “Disease or Syndrome” was mapped to the observation category with a probability of 79.89%. We used the training data to automatically create these heuristics. We used all (21) semantic types with a mapping probability of over 70%. The output semantic type was converted to a family member or observation entity, such as B-family_member or I‑observation.

Our Bi-LSTM model incorporated 2 embedding layers for pretrained word embeddings. We used dependency-based embeddings by Komninos and Manandhar [27] as static word embeddings. These embeddings were trained using the structure of dependency graphs. They were built with the English Wikipedia Dump of August 2015. As context-dependent embeddings, we used the ELMo [21] model trained on a dataset of 5.5 billion tokens from Wikipedia and the news crawl corpus. The output of each external resource (the 2010 i2b2 and MetaMapLite) was represented as a one-hot vector and mapped to a 10-dimensional embedding. The concatenation of these embeddings (2 pretrained embeddings and 2 one-hot vectors) was fed to the LSTM layer.

To fine-tune the parameters of LSTM models, we randomly selected 10 documents from the training set (about 10% of the training set) as held-out data. We tuned the hyperparameters to maximize the F_1_ score with the held-out data. After experimenting with different dropout [28] rates of 10%, 20%, 30%, 40%, and 50%, the models were trained using the Nadam [29] optimizer for 30 epochs with a dropout rate of 50%.

**Table 2:** The 10 most frequent Unified Medical Language System (UMLS) semantic types aligned with labeled observations in the training set.

Semantic type name	Abbreviation	Count	Probability, %
Disease or syndrome	dsyn	433	79.89
Neoplastic process	neop	165	78.20
Mental or behavioral dysfunction	mobd	59	74.68
Sign or symptom	sosy	28	70.00
Congenital abnormality	cgab	27	90.00
Anatomical abnormality	anab	10	83.33
Body system	bdsy	8	72.73
Tissue	tisu	7	100.00
Cell	cell	5	83.33
Physiologic function	phsf	4	80.00

We trained 10 different Bi-LSTM models that use the same hyperparameters but differ in random weight initialization and shuffling of training data. Then, we created a voting ensemble method that combined the predictions of all Bi-LSTM trials. Although these LSTM models were trained with the same hyperparameters, we hypothesized that they can be contributory to the voting ensemble in terms of diversity. Reimers and Gurevych [30] showed that nondeterministic LSTMs can even lead to statistically significant differences between multiple runs.

The voting ensemble collected candidate entities that received more votes than the voting threshold. When there were overlapping text spans on 2 different entities, the entity with more votes was selected. For overlapping entities with the same vote count, the one produced by the higher-ranking model was selected. To determine the ranking of 10 individual models, we measured how each model agreed with the other 9 models. Rankings were based on F_1_ scores measured with other models. The higher the average F_1_ score, the higher the model ranking.

#### Heuristic Rules for Family Member Attributes

We assigned each family member entity a normalized form using a simple dictionary-based mapping. For example, a family member with the text “*his dad*” was assigned ‘*Father.*’ We changed the text to lower case and removed the numeric values (eg, *“three uncles*” becomes '*Uncle*'). We also looked at the preceding words to search for another family member term that modified the target entity. When such a term was found, normalization was performed taking it into account. For example, in the phrase “*mother has sister,*” the family member ‘*sister*’ was normalized to ‘*Aunt.*’

Our rule-based system looked at words in sentences near the family member and considered the degree of relatives to determine the family side. For each family member who was not a first-degree relative, the side of family (ie, ‘*Paternal*’ or ‘*Maternal*’) was assigned. For each label, we compiled the list of cue words indicating the side of family. For example, the cues for *Paternal* included ‘*paternal,*’ ‘*patient's father,*’ ‘*father had,*’ and ‘*paternal family history.*’ First, we searched for cue words within the entity term itself. If no cue word was found, the search was expanded to sentence boundaries.

### Relation Extraction Methods

Subtask 2 aimed to identify related pairs of 3 entity types: family members, observations, and living status. Two types of relation exist between the 2 entities: family member-living status relations and family member-observation relations. We trained 2 relation extraction models. The first model determined the living status of each family member. The second model identified observations associated with each family member.

For 2 binary-class models, we defined lexical features: words contained in each concept, 7 preceding and 7 following words for each concept, and the words between the 2 concepts. We also created 1 feature to measure the number of family member entities appearing between the pair. We created 2 binary-class OGD (also called stochastic gradient descent) [22] classifiers using the Vowpal Wabbit [31] online learning library. This online learning algorithm is getting more attention recently in large-scale machine learning problems. Using the default hyperparameters, each model was trained for 100 iterations.

Training examples included positive examples (participating in a relation) and negative examples (pairs of entities that are not related to each other). Pairs of reference standard entities were used to train the classifiers. Entity pairs identified by the aforementioned voting ensemble were used as test examples. We filtered out the negative examples when there was a carriage return character (‘\n’) between the pair.

For living status relations, once we extracted phrases that represent the living status of each family member, we assigned scores for the *alive* and *healthy* attributes. We compiled *not alive* (ie, dead) and *healthy* cues from the training data and calculated the score using the text phrase of each living status entity. If our algorithm detected any trigger phrase of *not alive* (eg, “*deceased,”* “*passed away*,” and “*no longer living*”), the algorithm assigned a score of 0. Otherwise, if the family member was in good health (eg, “*good general health*,” “*healthy*,” and “*alive and well*”), the algorithm assigned a score of 4. If no cues of *not alive* or *healthy* were found, a score of 2 was assigned.

For each observation entity in the relation, we needed to determine whether it was negated or not. We used FastContext [32], an efficient and scalable Java implementation of the ConText algorithm [33] with customized trigger terms. After manually analyzing the examples from the training data, we added new trigger terms such as “*not aware of,*” “*not significant*,” and “*no family history of*.” For this binary classification, the algorithm detected the negated contextual attribute in the sentence for the observation entity and assigned 1 of 2 values: *Negated* or *Non_Negated*.

**Figure 1:**
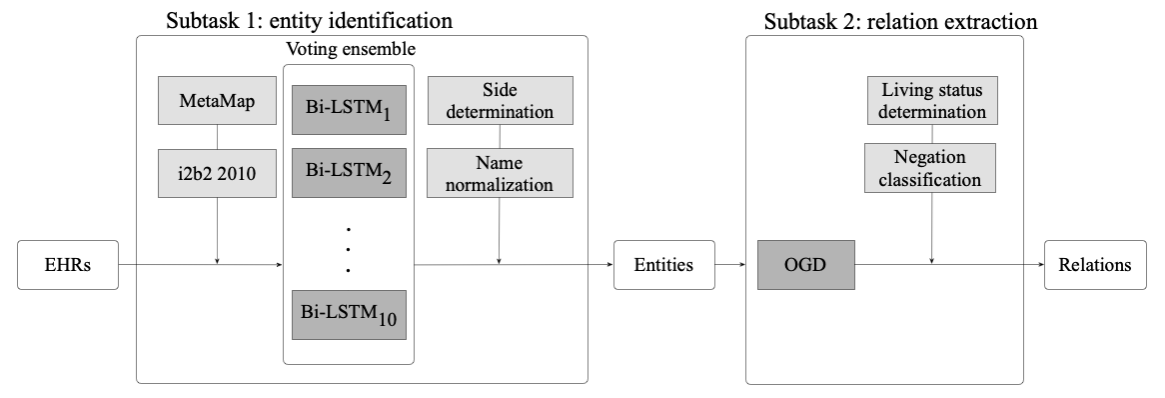
End-to-end system architecture. Bi-LSTM: bidirectional long short-term memory; EHR: electronic health record; i2b2: Informatics for Integrating Biology and the Bedside; OGD: online gradient descent.

In summary, we built an end-to-end system with multiple IE and attribute classification components, as shown in Figure 1. The architecture includes a voting ensemble with Bi-LSTM models that accept the outputs of the MetaMap and 2010 concept models, an OGD model that extracts relations between entities, and postprocessing modules for family side, name normalization, living status, and negation classification.

### Improvements to Both Subtasks After the Shared Task Challenge

This subsection describes further improvements to both entity identification and relation extraction as postchallenge efforts. We made 2 major changes in the pipeline system. The first revision was the addition of labeled examples to the training data. We used another text collection created for the 2018 BioCreative/OHNLP shared task [7] to build new Bi-LSTM and OGD models. The combined dataset included the original 99 clinical notes and 50 text files used in the 2018 BioCreative/OHNLP test set. Extending from the previous models used for submission to the shared task, we investigated how well the new model trained with the union of 2 datasets performed. We trained the new models by reusing the classifier configuration optimized with the 2019 training data.

Next, we used word embeddings trained with clinical text to construct vector representations of words. We pretrained 2 language models. One was trained using fastText [34] as static word embeddings, and the other was trained using ELMo [21] contextual embeddings. We used all clinical notes from the Medical Information Mart for Intensive Care (MIMIC-III) clinical database (version 1.4) [35]. We pretrained ELMo embeddings by following the default hyperparameter setting used for other publicly available ELMo models [21]. Pretraining lasted about 3 months, and it was manually stopped after 1,073,750 iterations. This process was performed on a NVIDIA Tesla P4 GPU.

From these pretrained language models, we generated word vectors as input features. Then, we created new Bi-LSTM models for entity identification. As with the previous models, these models were trained for 30 epochs with 50% dropout to the recurrent units. Naturally, the predictions of these new Bi-LSTM models were used to create test instances that paired the 2 entities for relation extraction. In the next section, we present the experimental results from our official submission and revised systems.

## Results

The input for subtask 1 (entity identification) was clinical text notes. The entity annotation file for subtask 1 contains family member and observation entities, one entity per line. The family side is provided for each family member entity. For subtask 2 (relation extraction), entity annotations were additionally used as input. The relation annotation file for task 2 contains 2 entities with their relation, 1 relation per line. Each living status relation has a score to represent living status. In each observation relation, the negation of the observation entity was identified.

### Evaluation Metrics

We measured recall, precision, and F_1_ score (harmonic mean of recall and precision with equal weight). We used the 2019 n2c2/OHNLP shared task [6] evaluation script to calculate performance measures. To be considered a true positive, the entity attributes must also match. For observation entities, a match was counted if the reference annotation contained 1 or more words in common with the system-detected concept.

### Results for the 2019 n2c2/OHNLP Shared Task

The 2019 n2c2/OHNLP shared task corpus consisting of a test set of 117 clinical notes was used for the evaluation. First, we present the results generated by systems implemented for the 2019 n2c2/OHNLP shared task submission.

Table 3 shows the microaveraged overall precision, recall, and F_1_ score for each of our submissions. The following 3 systems were submitted for subtask 1: System 1.1 was a rule-based system for collecting family member entities and a voting ensemble with a voting threshold of 5 for extracting observation entities, system 1.2 was a voting ensemble consisting of 10 trials with a voting threshold of 5 for extracting family member and observation entities, and system 1.3 was a voting ensemble with a voting threshold of 6. Among them, system 1.2 achieved the highest F_1_ score, 81.30%, in subtask 1.

**Table 3:** Results produced for the 2019 National Natural Language Processing Clinical Challenge (n2c2)/Open Health Natural Language Processing (OHNLP) shared task.

System		Precision score	Recall score	F_1_ score
**Subtask 1 (entity)**				
	System 1.1	72.61	86.01	78.74
	System 1.2	78.90	83.84	81.30
	System 1.3	80.29	81.98	81.13
**Subtask 2 (relation)**				
	System 2.1	65.48	64.41	64.94
	System 2.2	66.37	62.78	64.53
	System 2.3	68.23	59.79	63.73

Similarly, we submitted 3 systems for subtask 2: System 2.1 was an OGD model with input pairs generated from predictions of the voting ensemble with a voting threshold of 4, system 2.2 was an OGD model with outputs from system 1.2, and system 2.3 was an OGD model with outputs from system 1.3. System 2.1 achieved a higher F_1_ score than the others. The range of vote thresholds for task submission was selected after experimenting with values from 1 to 10 on the validation set. The highest F_1_ score was obtained in subtask 2 with a voting threshold of 5 on the validation set.

### Improved Results After the Shared Task

We report the results of further improvements for both subtasks as described earlier. The contributions of features or data are shown in Table 4. Systems from rows 1 to 3 were developed for the 2019 n2c2/OHNLP challenge, and rows 4 and 5 were postchallenge efforts.

**Table 4:** Improved performance by feature or data accumulation.

System		Precision score	Recall score	F_1_ score
**Subtask 1 (entity)**				
	(1) word	78.50	84.28	81.26
	(2) + MetaMap, i2b2^a^ 2010	78.87	84.34	81.48
	(3) + voting	78.90	83.84	81.30
	(4) + 2018 data (postchallenge)	83.63	86.69	85.13
	(5) + MIMIC^b^ embeddings (postchallenge, [2018 + 2019]_mim_)	84.83	87.24	86.02
**Subtask 2 (relation)**				
	(1) word	65.35	61.14	63.14
	(2) + MetaMap, i2b2 2010	66.34	61.24	63.66
	(3) + voting	66.37	62.78	64.53
	(4) + 2018 data (postchallenge)	72.15	70.79	71.46
	(5) + MIMIC embeddings (postchallenge, [2018 + 2019]_mim_)	73.27	71.70	72.48

^a^i2b2: Informatics for Integrating Biology and the Bedside.

^b^MIMIC: Medical Information Mart for Intensive Care.

As a baseline, only sequences of word tokens were used as input to train the Bi-LSTM models (row 1). The system was enhanced with the output of MetaMapLite [26] and the 2010 i2b2 [25] concept model as inputs (row 2). For rows 1 and 2, we report the average value between the 10 trials of each Bi-LSTM model. From row 3, the results of applying the voting ensemble are displayed. For comparison, we report results with a voting threshold of 5. Row 4 shows a further performance improvement when the 2018 BioCreative/OHNLP shared task [7] data were added. This additional training example achieved substantial performance improvements in both subtasks. Compared to the submission for the challenge (row 3), the recall increased by 8.01% (70.79%-62.78%) in subtask 2. MIMIC embeddings (row 5) allowed for an improvement over general text embeddings. They led to F_1_ scores of 86.02% and 72.48% for subtask 1 and subtask 2, respectively. We used a chi‑squared test to measure statistical significance. The significance level was set to .05. The performance of the full-featured system, called *(2018 + 2019)_mim_*, (row 5) was significantly better than other systems with *P* values <.001 except the system with the 2018 BioCreative/OHNLP shared task data (row 4).

Table 5 displays the precision, recall, and F_1_ scores of relation categories produced by the (2018 + 2019)_mim_ system. F_1_ scores for living status relations were 84.62% and 74.72% for subtask 1 and subtask 2, respectively. It was more challenging to determine whether the pair of family member and observation was related. For observation relations, the F_1_ score was 71.79%, which was lower than for living status relations. A manual analysis of labeled examples from the training set revealed that distant pairs of family member and observation appeared more often than living status entities. In addition, there were more unrelated entity pairs (ie, negative examples) because many observation entities were not involved in the relation.

**Table 5:** Results of full-featured system for each relation category.

System		Precision score	Recall score	F_1_ score
**Subtask 1 (entity)**				
	Living status	83.08	86.21	84.62
	Observation	85.99	87.92	86.94
	Overall	84.83	87.24	86.02
**Subtask 2 (relation)**				
	Living status	73.28	76.22	74.72
	Observation	73.27	70.37	71.79
	Overall	73.27	71.70	72.48

## Discussion

The experimental results show that our end-to-end pipeline system substantially benefited from the combination of the 2 datasets. Another finding is that a voting ensemble could achieve better performance than individual classifiers. This section analyzes the improvements resulting from the voting ensemble method. We also describe the detailed results of attribute classification.

### Voting Ensemble Analysis

**Figure 2:**
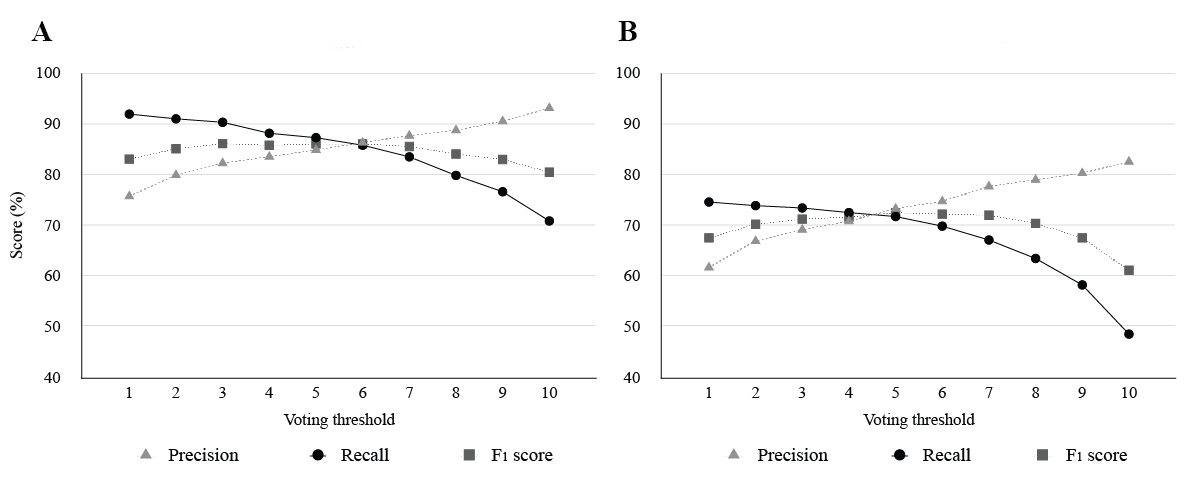
Results of the voting ensemble for (A) subtask 1: entity identification and (B) subtask 2: relation extraction.

We analyzed the performance of the voting ensemble at each voting threshold. Figure 2 shows the results of the voting ensembles with 5 trials of the (2018 + 2019)_mim_ system. The graphs on the left and right represent the results of subtask 1 and subtask 2, respectively. The y-axis scale of each graph does not start at zero to focus on the value ranges of interest. The results with voting thresholds ranging from 1 to 10 are presented. The curves show that as the threshold gets higher, precision increases but recall simultaneously decreases. When the threshold was set to 3, the ensemble achieved the highest F_1_ score (86.07%) in subtask 1. For subtask 2, the ensemble obtained an F_1_ score of 72.48% at the voting threshold of 5.

### Attribute Classification Analysis

We applied heuristics to determine the attributes of entities. As the entity-level reference standard in the test set was being withheld, we evaluated the performance of these rule-based methods on the training set. Table 6 shows the accuracy of the 4 classification tasks with the given reference standard concepts. Accuracy was computed as the percentage of correct predictions among total instances. The accuracy of family member normalization was 94.01%. Our classifier rarely failed to assign normalized terms to some entities. For example, our dictionary did not contain normalized terms for “*twin*” and “*paternal relatives.*” Most errors occurred when the normalized term did not match the actual relationship with the patient. For example, although it said “*brother*” in the text, it sometimes referred to the relationship with the patient’s parents, not the patient himself. The classifier often could not determine the family member as the patient's “*Uncle.*” This type of error was propagated in family-side decisions because the family-side information should only be provided to first-class relatives.

**Table 6:** Accuracy of attribute classification of given reference standard concepts.

Task	Accuracy (%)
Normalization of family members	94.01
Determination of the side of family	95.38
Assessment of family member’s living status	92.53
Detection of negation information for observations	98.06

In addition to the entity-level assessment described in the previous paragraph, we conducted another document-level evaluation of the entity attributes against the test set. To measure the performance impact of each attribute classification, the system was tested by ignoring one attribute of the entity. Table 7 shows the results of the 2 subtasks on the test set by the (2018 + 2019)_mim_ system. We report results for living status and negative information only for subtask 2 because they are not considered in subtask 1. A match is made if the system correctly detects an entity while the attribute is ignored. Compared to the default evaluation, which considered all attributes, it led to higher values for all metrics. Ignoring living status scores had the biggest impact. If the living status of every family member was correctly determined, the F_1_ score could be increased by about 2%. Negative information had the least impact because it only applied to observations and might have been determined more accurately than other attributes.

**Table 7:** Performance impact of attribute classification.

System		Precision score	Recall score	F_1_ score
**Subtask 1 (entity)**				
	Default evaluation	84.83	87.24	86.02
	Ignoring the side of the family	85.92	89.11	87.49
	Ignoring the living status	N/A^a^	N/A^a^	N/A^a^
	Ignoring negation	N/A^b^	N/A^b^	N/A^b^
**Subtask 2 (relation)**				
	Default evaluation	73.27	71.70	72.48
	Ignoring the side of the family	75.33	73.64	74.48
	Ignoring the living status	75.40	73.78	74.58
	Ignoring negation	73.85	72.90	73.37

^a^N/A: not applicable as the living status information was removed from evaluation for subtask 1.

^b^N/A: not applicable as the negation information was removed from evaluation for subtask 1.

### Limitations

We observed in this study that determining the voting threshold can be challenging for both subtasks. Our results showed that the best performing voting ensemble for one task did not achieve the highest accuracy for the other task. More efficient ensemble approaches will be desired to provide more diversity between individual models and reduce the error rate through optimal control of agreements among them. In the relation extraction task, the negative examples were filtered out when there was a carriage return character between the pairs, because they rarely appeared in the training data (about 2.6%). This instance pruning would make it impossible to find pairs of entities that existed in different sentences but were related. When training new models by combining 2 corpora, we reused the classifier configuration optimized for the 2019 n2c2 model. New development data randomly selected from both corpora would be needed for hyperparameter tuning.

### Conclusions

We presented a hybrid method that combined machine learning and rule-based approaches developed as part of the 2019 n2c2/OHNLP track on FH extraction [6]. The MUSC team ranked 3rd and 4th among the participating teams in subtask 1 and subtask 2, respectively. This study demonstrated that our end-to-end pipeline system could successfully extract FH information recorded in unstructured narrative free text. Our experimental results confirmed that the voting ensemble of multiple trials outperformed the individual classifiers that produced nondeterministic results. Our postchallenge efforts significantly improved performance by leveraging additional labeled data and using word vector representations learned from large collections of clinical notes.

Further research includes creating machine learning–based classifiers that will replace rule-based systems that determine the attributes of entities. They could lead to more accurate results on attribute classification as reported in several studies carried out for similar clinical NLP tasks [36-39]. Another direction for future work is to exploit unlabeled data to collect texts from the family history section. For efficient extension of the amount of training data, semisupervised learning can be employed with an instance selection method that uses text similarity measures to consider representativeness and diversity [40].

## Abbreviations

Bi-LSTM: bidirectional long short-term memory
EHR: electronic health record
ELMo: embeddings from language models
FH: family history
i2b2: Informatics for Integrating Biology and the Bedside
IE: information extraction
LSTM: long short-term memory
MIMIC: Medical Information Mart for Intensive Care
MUSC: Medical University of South Carolina
n2c2: National Natural Language Processing Clinical Challenges
NER: named entity recognition
NLP: natural language processing
OGD: online gradient descent
OHNLP: Open Health Natural Language Processing
UMLS: unified medical language system
